# Non-periodized and Daily Undulating Periodized Resistance Training on Blood Pressure of Older Women

**DOI:** 10.3389/fphys.2018.01525

**Published:** 2018-11-27

**Authors:** Hélio J. Coelho-Júnior, Ivan de Oliveira Gonçalves, Niels O. S. Câmara, Marco A. Cenedeze, Reury F. Bacurau, Ricardo Yukio Asano, Jeferson Santana, Erico Caperuto, Marco C. Uchida, Bruno Rodrigues

**Affiliations:** ^1^Applied Kinesiology Laboratory, Department of Adapted Physical Activity, Faculty of Physical Education, Universidade Estadual de Campinas - UNICAMP, Campinas, Brazil; ^2^Exercise Physiology Applied to Disease Research Group, Department of Adapted Physical Activity, Faculty of Physical Education, Universidade Estadual de Campinas - UNICAMP, Campinas, Brazil; ^3^Community Center for Older People of Poá, Poá, Brazil; ^4^Laboratory of Transplantation Immunology, Department of Immunobiology, Institute of Biomedical Sciences, University of São Paulo, São Paulo, Brazil; ^5^Nephrology Division, Federal University of São Paulo, São Paulo, Brazil; ^6^School of Arts, Sciences and Humanities, University of São Paulo, São Paulo, Brazil; ^7^Human Movement Laboratory, Universidade São Judas Tadeu, São Paulo, Brazil

**Keywords:** physical exercise, strength training, power training, periodization, hypertension, blood pressure, elderly, nitric oxide

## Abstract

The present study aimed at investigating the effects of a daily undulating periodization (DUP) and non-periodized (NP) resistance training programs on hemodynamic parameters of older women. Forty-two older women were randomized into one of the three experimental groups: NP, DUP, and control group (CG). Evaluations of the hemodynamic parameters occurred before, during and after the intervention. The exercise programs were performed twice a week over 22 weeks. NP and DUP groups were based on 3 sets of 8–10 repetitions in 9 exercises. In NP, the two exercise sessions were based on traditional strength training, which was performed at a Difficult intensity according to the rating of perceived exertion (RPE) method. In DUP, the first session was based on power resistance exercise, in which the concentric muscle contraction was performed as fast as possible at a moderate intensity based on RPE, while the second session was the same that was performed by NP. The findings demonstrated that diastolic blood pressure (90.4 vs. 76.2 mmHg) and mean arterial pressure (108.6 vs. 92.7 mmHg) were significantly reduced after NP, while no significant alterations were observed in DUP. Nevertheless, both training groups seem to have a cardio protective effect, since both training modes prevented the increase in HR reported in the experimental period in CG. In conclusion, our findings indicate that a 22-week NP resistance training program causes beneficial effects on hemodynamic parameters of older women.

**Trial Registration:** NCT03443375.

## Introduction

Hypertension (HTN) is one of the most prevalent diseases in older people, given that more than 70% of those aged 60 years or more around the world have been clinically diagnosed with this condition (Mozaffarian et al., 2015). The progression of this disease is certainly a public health problem since patients with high blood pressure show increased cardiovascular risk (World Health Organization, 2009; Mozaffarian et al., 2015). Regarding gender prevalence, women and men are both highly affected by HTN (Mozaffarian et al., 2015). However, menopause and aging process have a prominent role in the development and progress of HTN in women, which makes HTN prevalence higher in women than in men during the sixth decade of life and over (Mozaffarian et al., 2015; Wenger et al., 2018).

Before menopause, sex hormones act directly and indirectly in the homeostasis of the cardiovascular system by controlling several physiological processes, including vascular control (Qiao et al., 2008; Wenger et al., 2018). In relation to the last, sex hormones mediate vascular control through their binding to vascular receptors causing endothelium-dependent vasodilatation by a mechanism dependent of nitric oxide (NO) (Qiao et al., 2008). In contrast, this phenomenon is impaired during menopause and aging in response to reduced levels in sex hormones (Qiao et al., 2008). These changes on NO pathway deserve concern because NO availability is strongly associated with cardiovascular control and functioning (Farah et al., 2018).

HTN management is a public health problem and drastic changes in lifestyle have been widely recommended for hypertensive patients, regardless of HTN classification (Chobanian et al., 2003). It should be stressed, that resistance training (RT) has been highlighted among the lifestyle changes and suggested as a non-pharmacological therapy because evidence indicates that this kind of exercise may control blood pressure alone in normotensive and hypertensive subjects (Moraes et al., 2012; Mota et al., 2013; de Sousa et al., 2014; Tomeleri et al., 2017; Terra et al.). The RT prescription allows innumerable combinations among the variables of exercise training. In the context of HTN, numerous studies have investigated traditional strength training (ST) (Moraes et al., 2012; Tomeleri et al., 2017), which is commonly based on moderate-to-high loads (e.g., 60–80% of 1 repetition maximum [1RM]), short-time intervals (e.g., 1–2 min), and muscular contractions that last from 2 to 3 s (Kraemer and Ratamess, 2004; Chodzko-Zajko et al., 2009).

Although some evidence has demonstrated reduced blood pressure values after traditional ST programs, an apparent paradox seems to be associated with this kind of exercise training. In fact, if, on the one hand, exercise intensity seems to be crucial to the beneficial effects elicited by ST (Figueiredo et al., 2015); on the other hand, blood pressure increases to undesirable levels during ST accordingly to training load and muscle failure (MacDougall et al., 1985b). Therefore, one question that remains is what the best design of ST to cause reductions in blood pressure, while the individuals are submitted to a low cardiovascular stress. In this sense, the investigation of different models of organization and designs of ST seems to be necessary and may collaborate to clarify the insufficient knowledge about the prescription of RT on hypertensive patients.

As for the organization models of ST variables, it is possible to suggest that exercise periodization may be an advantageous method to prescribe ST in an attempt to promote cardiovascular benefits. Periodized exercise training has been used since ancient Greece, where Philostratos proposed that Olympic athletes should divide their training routine in short cycles devoted to improving different physical capabilities (Grivetti and Applegate, 1997). Nowadays, periodization is characterized as an approach that allows the organizing of exercise training variables, typically training intensity and training volume, into sequential phases and cyclical time periods (Issurin, 2010; Williams et al., 2017).

During the past years, some periodization methods have been proposed and tested for effectiveness, mainly for neuromuscular adaptations (Issurin, 2010; Williams et al., 2017). However, only the effects of traditional, or also called linear periodization model, in which an inverse relationship is observed between ST volume and intensity over the course of the exercise program, have been investigated in the cardiovascular context (Faria Terra et al., 2008; Mota et al., 2013; Moreira et al., 2016). Nonetheless, when individuals are submitted to a ST program based on linear periodization model they will experience short- (microcycle) and medium-term (mesocycles) periods of ST in which the load will remain markedly elevated (≥80% 1RM), limiting the clinical applicability of this kind of program.

It is worth mentioning, that daily undulating periodization (DUP) is one periodization design that has not been well-investigated in the context of cardiovascular system, but has demonstrated numerous beneficial effects on the neuromuscular system when compared to non-periodized (NP) and traditional models of periodization (Rhea et al., 2002; Simão et al., 2012). DUP is classically characterized by marked changes in the training phases within a microcycle (Rhea et al., 2002; Simão et al., 2012); thus, if a session of moderate-to-high intensity ST was performed in the first session, a session at low-to-moderate intensity should be performed on the next training day. Consequently, this periodization design may be an alternative to the traditional model and seems to be attractive for older adults, mainly hypertensive patients, because a higher cardiovascular stress is not expected during its performance. Nevertheless, DUP is not free of certain problems, because the session performed at a lower intensity may be ineffective to elicit cardiovascular improvements and, consequently, blood pressure reduction.

Recently, our group demonstrated that a session of light-to-moderate intensity power resistance exercise, another type of RT, causes a significant reduction on blood pressure values in hypertensive older women (Coelho-Júnior et al., 2017a). These findings seem to be interesting, as power resistance exercise is a well-tolerated, non-fatiguing regime of RT performed at an explosive lifting velocity at light-to-moderate loads, with submaximal repetitions (Izquierdo and Cadore, 2014; Cadore and Izquierdo, 2018). All these features make power resistance exercise a secure and effective option to DUP programs. In fact, as the cardiovascular responses to power resistance exercise seem to be dependent on the velocity of concentric muscle contraction, regardless exercise intensity (Coelho-Júnior et al., 2017a; Coelho Junior et al., 2017b), it can be an excellent alternative to the lower exercise session of DUP. In addition, a lower cardiovascular stress may be expected during power resistance exercise performance, since it does not include high training loads, fatiguing exercises, and Valsalva maneuver, all variables associated with desirable increases on blood pressure during exercise (MacDougall et al., 1985b).

Therefore, we hypothesized that combine traditional RT and power resistance exercise in a DUP program may be an effective strategy to induce similar or even greater decreases in blood pressure when compared to NP ST, while the subjects are submitted to a low cardiovascular stress. In this sense, the present study aimed at investigating the effects of a DUP program—composed by ST and power resistance training (PT)—and NP ST on hemodynamic parameters and salivary NO availability of older women.

## Materials and methods

### Experimental design

This is a randomized clinical trial (ClinicalTrials.gov Identifier: NCT03443375). Volunteers were randomized and allocated into one of the three experimental groups: NP, DUP, and control group (CG). The exercise protocols were based on the American College of Sports and Medicine (ACSM) guidelines (Chodzko-Zajko et al., 2009; Garber et al., 2011). The current investigation was carried out over a total period of 26 weeks. The first and last 2 weeks were dedicated to evaluations, which occurred with volunteers fasted (at least 12 h), abstained from caffeine and alcohol, and having refrained from exercise for at least 96 h prior to testing. Therefore, NP and DUP groups performed the exercise sessions over 22 weeks.

### Subjects

**Figure 1:**
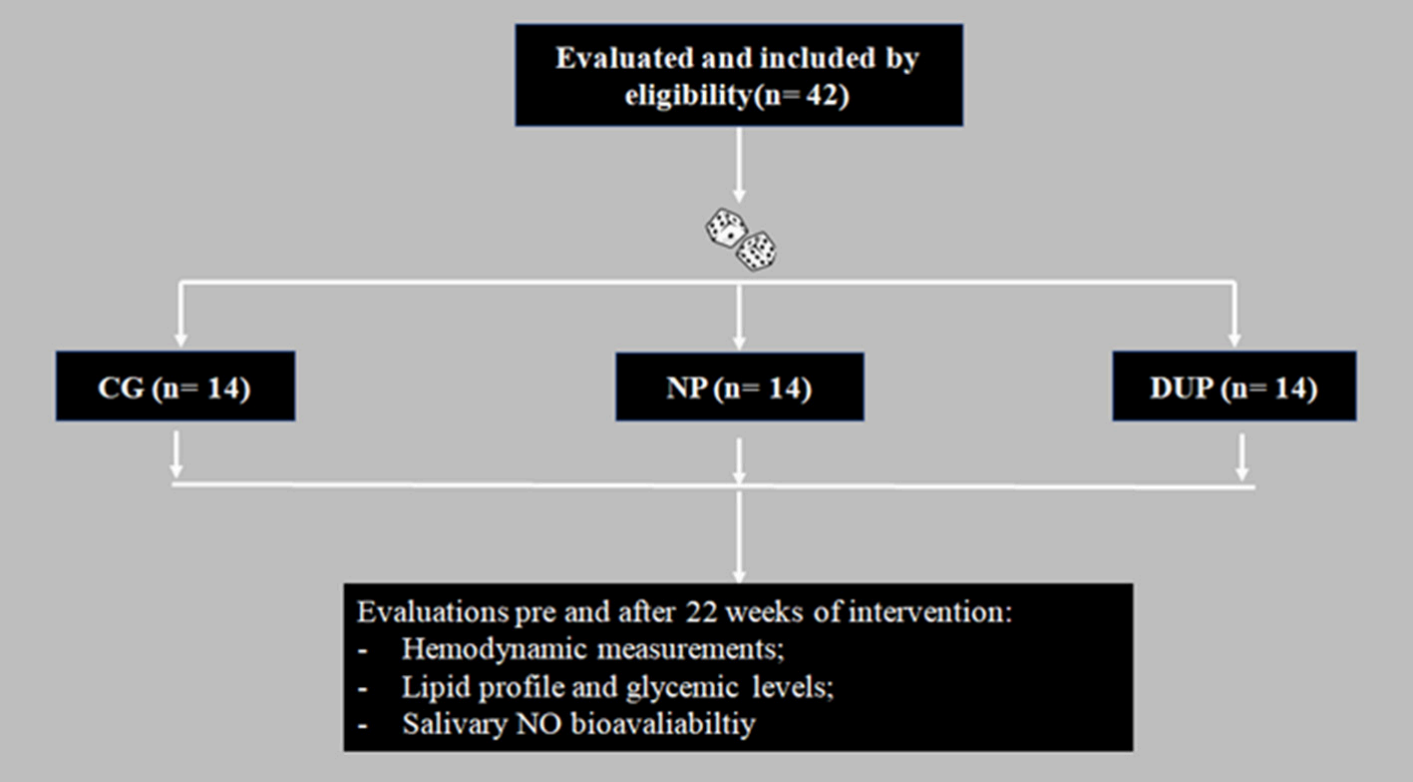
Flowchart of the study. NP, Non-periodized; DUP, Daily undulating periodization; CG, Control group; NO, Nitric oxide.

Forty-two untrained community dwelling-older women (age range 60–74) volunteered to take part in the present study. In order to be included in the study, participants had to be females, ≥60 years, post-menopausal by at least 1 year, be diagnosed with stage I HTN (Chobanian et al., 2003), and capable of attending all training and testing sessions. Patients who dropped out from the intervention, presented changes on antihypertensive medication in the past 6 months prior to inclusion in the study, a clinical diagnosis of resistant HTN, cardiovascular (i.e., acute myocardial infarction, stroke, peripheral arterial disease, and transient ischemic disease), metabolic (i.e., diabetes mellitus type I or II), pulmonary disease (i.e., emphysema), neurological and/or psychiatric disease (i.e., Parkinson's or Alzheimer's disease), skeletal muscle disorders, comorbidities associated with greater risk of falls, recent history of smoking or alcohol abuse, and engagement in physical exercise training programs (three sessions per week) in the past 6 months prior to inclusion (untrained), in the study were excluded. We also excluded participants who were prescribed hormone replacement therapy and/or psychotropic drugs. Prior to any evaluation, a physician authorized volunteers to participate in the trial. In addition, diagnosis of HTN was not a criterion for exclusion. However, to ensure homogeneity, hypertensive, and normotensive subjects were randomly allocated into NP, DUP and CG groups. In this sense, experimental groups were composed of 50% of hypertensive patients and 50% of normotensive subjects. A computer-generated list of random numbers was used for allocation of the participants into one of the 3 experimental groups (i.e., NP, DUP, and CG) (Figure 1). A researcher, who was blinded to the characteristics of subjects, performed the randomization before baseline evaluations. Volunteers gave written informed consent according to the Helsinki protocol before entering the study, which was approved by the Research Ethics Committee of the University of Campinas.

### Procedures

#### Exercise protocols

During all procedures, including physical training, the temperature in the laboratory was maintained between 21 and 24°C. Volunteers did not receive dietary recommendations. However, they were instructed not to change their dietary habits and activities of daily living during the entire study period. The exercise protocols occurred under the supervision of three experienced researchers.

#### Exercise training programs

**Figure 2:**
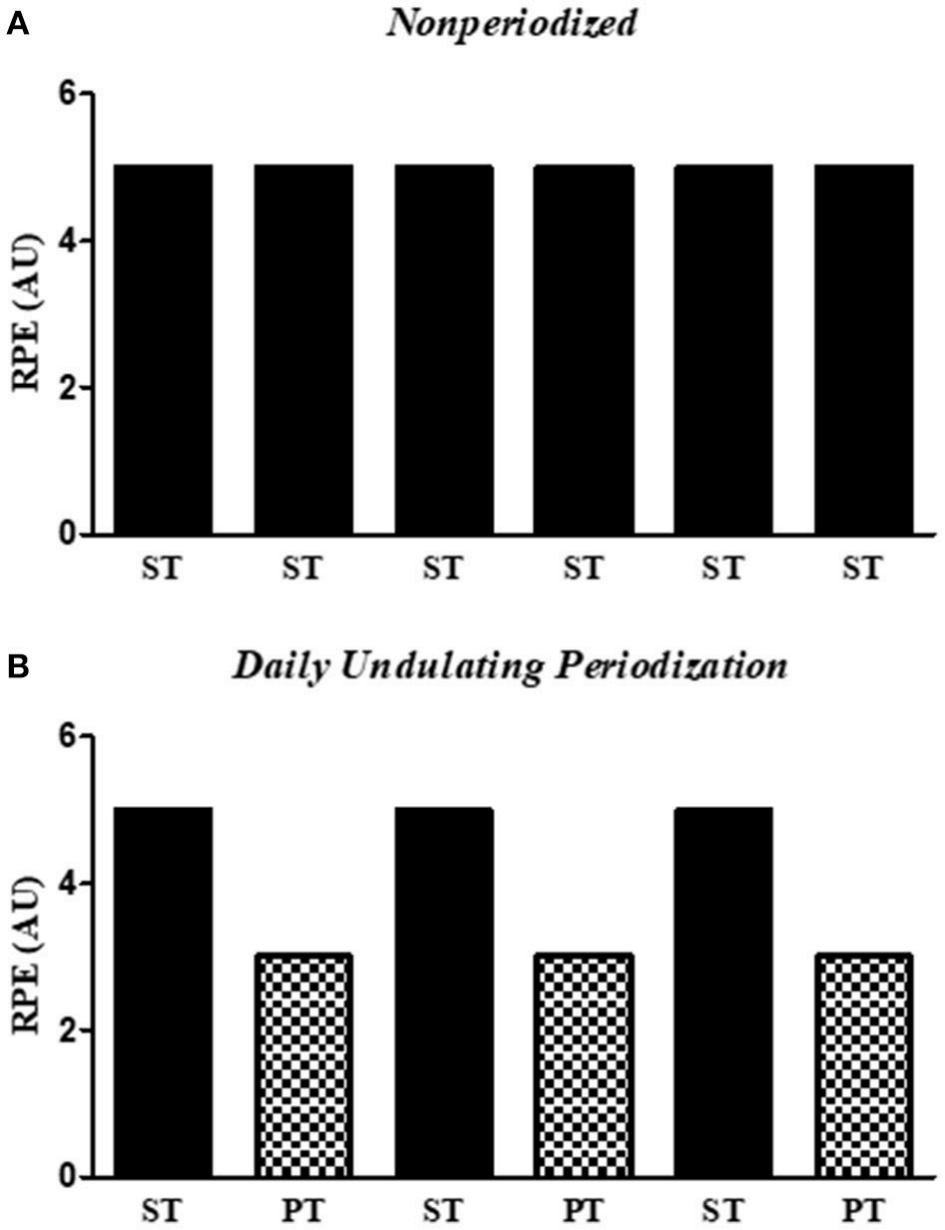
Training load distribution in the Non-periodized **(A)** and Daily undulating periodization **(B)** programs over 3 weeks. It is possible to observe that the training load remains constant across the non-periodized program, while it is distributed according to a wave shape in the daily undulating periodization program. RPE, Rating of perceived exertion; AU, Arbitrary units; PT, Power training; ST, Strength training.

The current investigation was carried out over a total period of 26 weeks. However, the two initials and the two last weeks were dedicated to evaluations. The exercise program was divided into two periods: (1) 4-weeks familiarization period and (2) 18-weeks main exercise period, totalizing 22 weeks. In both familiarization and main exercise periods, training sessions occurred twice a week, at a regular time of the day (08:30 a.m.−11:30 a.m.), with a minimum interval of 48 h between each exercise bout, under the supervision of three exercise physiologists. The program of exercise was individualized and conducted in pairs, which was accompanied by an exercise physiologist. The familiarization period consisted of nine exercises aimed at exercising the major muscle groups using alternating upper- and lower-body exercises. The exercises were performed exactly in the following order: (1) seated row, (2) leg press, (3) chest press, (4) seated leg curl, (5) lateral arm raise, (6) calf raise, (7) arm curl, (8) rope pushdown, and (9) abdominal crunch. The number of sets was increased during the first month, given that one set was performed in the 1st week; two sets in the 2nd and 3rd weeks; and 3 sets in the 4th week. Exercises were performed with 12–15 submaximal repetitions avoiding fatigue (i.e., inability to complete a repetition in a full range of motion) at an easy intensity according to Rating of Perceived Exertion (RPE, CR-10), adapted Borg scale (Foster et al., 2001). The main exercise period was the same for NP and DUP. The volunteers performed the same exercises and the total volume (number of sets × number of repetitions) was equalized between exercise groups. This period also consisted of nine exercises performed three sets (times) of 8–10 submaximal repetitions with a 1-min rest interval period been provided between sets. The list of exercises was almost the same of the first month, session training A and B; Training A (e.g., Monday), the only difference of familiarization period was the order and two exercises (1) squat on the chair (90° knee flexion), (2) chest press, (3) seated leg curl, (4) seated row, (5) frontal arm raise, (6) calf raise, (7) arm curl, (8) rope pushdown, and (9) abdominal crunch; while in training B (e.g., Thursdays), volunteers performed the same list of exercises of familiarization period. All exercises were performed in the full range of motion and all groups performed a brief warm-up at the beginning of each session, which was based on one set of 12–15 repetitions at easy (i.e., 2) intensity (Foster et al., 2001). In addition, volunteers were instructed to avoid the Valsalva maneuver during the entire muscle contraction, regardless of the session of exercise. Exercise intensity and the velocity of concentric muscle contraction were modified differently for each group according to the peculiarities of each type of exercise, as recommended by the ACSM (Chodzko-Zajko et al., 2009). In this sense, the NP group performed the exercise sessions at a difficult (i.e., RPE = 5–6) intensity (Foster et al., 2001) using machines (Johnson Health Tech, EUA) and free weights. Exercise cadence was 2 seconds for concentric and eccentric phases. On the other hand, to offer a PT session (Cadore and Izquierdo, 2018) for DUP, concentric contractions were performed as fast as possible, while the eccentric phase was performed within 2 s during training A. In addition, all exercises were performed at a moderate (i.e., RPE = 3) intensity (Foster et al., 2001) using elastic bands (Thera Band, Ohio, USA). The training B in DUP was the same as that performed by NP (3 sets of 8–10 repetitions at a difficult intensity using machines and free weights). To offer to the volunteers a more favorable physiological environment, the exercise groups performed a regenerative week every 4 weeks (i.e., tapering). In this week, the sessions of exercise were based on 3 sets of 12–15 submaximal repetitions of each exercise, at easy (i.e., RPE = 2) intensity (Foster et al., 2001). Therefore, whether the subject reported a RPE below the programmed, the weight was increased 2–5% for the upper limb exercises and 5–10% for the lower limb exercises (Chodzko-Zajko et al., 2009). The design of each group across the intervention may be seen in Figure 2. Training load was adjusted based on the rating of RPE method, using the CR-10 (Foster et al., 2001).

#### Elastic bands

The elastic bands were used to offer to the volunteers the possibility to perform the concentric contraction as fast as possible, avoiding any range-of-motion limitations imposed by the machines, given that elastic bands propitiate large mobility in the achievement of the movement (Uchida et al., 2016). Elastic bands are portable, inexpensive, reliable, and have high practical application. Moreover, several studies have been showing similar muscular activations across the muscle contraction during single- and multiple joint exercises (Aboodarda et al., 2016), perceived loading (Andersen et al., 2010), and morphofunctional adaptations (Colado and Triplett, 2008) after RT programs with equalized exercise intensity performed with machines or elastic bands. Therefore, the elastic bands in the present study should be viewed as a tool to provide load and mobility, like machines and free weights, and not an independent variable. Volunteers of the present study performed the exercises using yellow, red and green elastic bands (Thera Band, Ohio, USA), which were kept tensioned across the entire muscle contraction, and any kind of slack in the elastics was largely avoided. All the elastic bands were warily used and then store according to the manufacturer's directions (Thera Band, Ohio, USA).

### Control group

The CG remained their regular habits of life during the entire study period, without engaging in physical exercise programs. To ensure that the volunteers did not engage a physical exercise program, face-to-face or telephonic contact was performed every 15 days.

### Evaluations

All subjects were instructed to refrain from physical exercise for 96 h before the tests. All tests were conducted between 07:00 am. and 10:00 am. under a controlled temperature of 26°C.

#### Hemodynamic measurements

In the morning of the experiment, upon arriving at the laboratory, the participants were asked about any basic needs (drink water or use the bathroom) before the beginning of the experiments and were urged to avoid doing these activities during the experiments. The procedures for measurement of blood pressure were adapted from the VII Joint National Committee on Prevention, Detection, Evaluation, and Treatment of High Blood Pressure (JNC7) (Chobanian et al., 2003). In summary, older women remained in a seated position on a comfortable chair for 20 min in a quiet room. After this period, an appropriate cuff was placed at approximately the midpoint of the upper left arm (heart level). An automatic, non-invasive, and validated (Cuckson et al., 2002) arterial blood pressure monitor (Microlife-BP 3BT0A, Microlife, Widnau, Switzerland) was used to measure systolic blood pressure (SBP), diastolic blood pressure (DBP), and heart rate (HR). During blood pressure recordings, volunteers remained relaxed in the sitting position, with parallel feet at shoulder width, both forearm and hands on the table, supinated hands, back against the chair, without moving or talking. The volunteer did not have access to blood pressure values during the measurement. The evaluation lasted approximately 80 seconds and was performed three times (the first measurement was discarded) with one minute of rest among the measurements. The test-retest reliability between the attempts was ≥0.8 (i.e., good reliability) according to Pearson's correlation coefficient. Mean arterial pressure (MAP), and double product (DP) were evaluated according to the following equations: MAP = [SBP + (2 ^*^ DBP)]/3, DP = SBP ^*^ HR. The size of the arm cuff was selected after measuring the arm circumference of each participant (Sanny, São Paulo, Brazil). All evaluations were performed in two non-consecutive days by the same investigator and the mean values of three measurements were used in the final evaluation.

#### Saliva collection

Saliva samples were collected at rest. Researchers asked the participants to put a piece of cotton in their mouth and remove it when it became soggy. The samples were transferred to a Falcon tube and frozen immediately until the end of the experiment. In the laboratory, the samples were centrifuged, and the supernatants were stored at −80°C for posterior analyses using the Griess colorimetric method (Coelho-Júnior et al., 2017a).

#### Measurement of nitric oxide (NO)

The NO pool was considered the mensuration of nitrite (NO${}_{2}^{-}$) levels in saliva. Briefly, a solution containing 0.1% N-(1-naphthyl)-ethylenediamine (NED) (Sigma) and 1.0% sulfanilamide (Sigma) was prepared in 2.5% phosphoric acid as the diluent. Saliva samples (50 μL) and Griess reagent (50 μL) were mixed and transferred to microplates. Absorbance was measured at 530 nm, and sodium nitrite (NaNO_2_) was used as the standard. Nitrite quantification (μM) was achieved using a standard curve constructed with NaNO_2_ at the concentrations of 100, 50, 25, 12.5, 6.25, 3.13, 1.66, and 0 μM. Data were analyzed using the Microplate software (CA, USA).

#### Lipid profile and fasting blood glucose levels

The biochemical variables measured were fasting blood glucose, triglycerides, total cholesterol, high-density lipoprotein (HDL) cholesterol, and low-density lipoprotein (LDL) cholesterol. Venous blood samples were collected by trained phlebotomists into sterile vacuum tubes, and plasma levels of the aforementioned lipid fractions and fasting blood glucose were measured using an ACON Laboratories (San Diego, CA, USA) and an SD Biosensor (Chungcheongkuk-do, SK) clinical chemistry systems, respectively.

#### Statistical analyses

Normality of data was tested using the Kolmogorov-Smirnov test. Baseline comparisons between the groups were performed using repeated measures one-way analysis of variance (ANOVA) followed by Tukey's posthoc test. An ANOVA [3 × 2, groups × time (pre- and post-22 weeks)] was used to examine differences between NP, DUP, and CG pre and post exercise, as the CG had only two measurement moments (pre and post). In addition, a second ANOVA [2 × 4, groups × time (Baseline, 4th week, 16th week, and 22nd week)] was used to examine differences between NP and DUP over the 4 measurement moments. In both cases, a Dunnet *post-hoc* test was performed to identify differences among the different times of evaluations and treatments. Cohen's d effect size was calculated to assess the magnitude of the results. The effect size (ES) was classified according to Rhea (2004) for untrained volunteers (i.e., trivial: < 0.50; small: 0.50–1.25; moderate: 1.25–1.9; large: >2.0). The level of significance was 5% (*P* < 0.05) and all procedures were performed using the GraphPad Prism 6.0. (San Diego, California, USA). The intention-to-treat principle was applied to the analysis of the outcomes for all participants based on their assigned treatment. Assuming a total withdrawal and dropout rate of 33% (6 volunteers), a level of significance set at 5%, power (β) of 0.80 and an effect size of 0.80, we estimated that a minimum of 24 older women (*n* = 8 per group) was required. However, due to convenience, 42 volunteers were recruited. The power analysis was performed using G^*^Power version 3.1.9.2. All values are shown as mean ± SD, except for ES that is shown as Cohens'd.

## Results

### Adverse events and training compliance

Subjects did not show adverse events during the sessions of exercise and during any evaluation. They were not absent for more than three sessions of physical exercise. Different compliance rates were observed among the groups, given that 4 participants dropout in the NP (*n* = 10; 28.5%), while 2 participants dropout in the DUP (*n* = 12; 14.2%). All volunteer reported personal reasons for leaving the protocol. The participation rate for NP and DUP groups were 88.7 and 90.9%, respectively. Finally, volunteers did not report any changes in food intake and in the number or class of medications during the whole course of the present study.

### Main characteristics

Table 1 shows the main characteristics of the volunteers before the beginning of the experiments. Body mass index (BMI) evaluation—according to specific cut-offs for older adults (Corona et al., 2015)—indicates that CG and DUP groups showed a normal phenotype, while NP showed an obesity phenotype. Blood pressure values in CG were classified as Prehypertension, while trained groups showed blood pressure values classified as Stage 1 Hypertension. However, as expected, the proportion of hypertensive patients was matched among the groups (50%). Data indicate that the pharmacological treatment of hypertensive patients was predominately based on angiotensin (ANG) II receptor antagonist−80% for all groups—and diuretics (CG = 10%; NP and DUP = 20%), followed by angiotensin-converting-enzyme (ACE) inhibitor (CG = 10%; NP = 20%) and calcium channel blockers, which was only used by DUP patients (10%). However, hypothesis tests did not indicate significant differences in any variable.

**Table 1:** Comparison between the groups in relation to morphological and cardiovascular variables.

	**Overall sample**			**Normotensive**			**Hypertensive**		
	**CG (*n* = 14)**	**NP (*n* = 10)**	**DUP (*n* = 12)**	**CG (*n* = 7)**	**NP (*n* = 5)**	**DUP (*n* = 6)**	**CG (*n* = 7)**	**NP (*n* = 5)**	**DUP (*n* = 6)**
Age (years)	68.4 ± 5.8	67.5 ± 4.4	66.7 ± 4.7	66.7 ± 4.0	66.6 ± 5.3	65.0 ± 2.3	70.1 ± 6.7	66.8 ± 7.7	68.5 ± 5.1
BMI (kg/m2)	25.2 ± 8.0	30.3 ± 4.02	25.6 ± 4.65	29.9 ± 3.8	31.5 ± 4.5	27.8 ± 6.0	20.5 ± 8.2	29.3 ± 2.3	27.8 ± 6.0
SBP (mmHg)	138.1 ± 13.7	145.1 ± 18.5	149.2 ± 22.5	128.0 ± 10.2	131.4 ± 14.6	129.0 ± 13.0	146.1 ± 8.7a	158.8 ± 9.9b	166.0 ± 12.8c
DBP (mmHg)	80.2 ± 11.6	90.5 ± 18.9	86.2 ± 8.6	76.1 ± 10.1	81.0 ± 6.0	84.6 ± 6.8	84.3 ± 11.5	100.0 ± 22.3	89.0 ± 8.4
MAP (mmHg)	99.4 ± 11.3	108.7 ± 16.3	107.7 ± 11.1	93.4 ± 8.8	97.8 ± 6.9	99.4 ± 8.6	104.9 ± 10.3	119.6 ± 15.7	114.7 ± 7.2
HR (bpm)	76.5 ± 7.6	78.1 ± 10.9	71.8 ± 10.1	78.4 ± 8.1	74.4 ± 7.5	71.0 ± 7.0	74.6 ± 6.3	81.8 ± 12.5	72.5 ± 12.1
DP (mmHg.bpm)	1.0512 ± 1.352	11356 ± 2380	1.0723 ± 2,208	10.021 ± 1.220	9.705 ± 921	9.237 ± 1.799	10.917 ± 1.287	13.006 ± 2.244	11.961 ± 1.694
Hypertension prevalence (%)	50	50	50	0	0	0	100	100	100
**DRUG CLASS (%)**									
ANG II receptor antagonist	80	80	80	—	—	—	80	80	80
ACE inhibitor	10	20	0	—	—	—	10	20	0
Diuretic	10	20	20	—	—	—	10	20	20
Calcium channel blockers	0	0	20	—	—	—	0	0	20

*Data are presented in mean ± SD.SBP, Systolic blood pressure; DBP, Diastolic blood pressure; MAP, Mean arterial pressure; HR, Heart rate; DP, Double product; NP, Non-periodized; DUP, Daily undulating periodization; CG, Control group; ANG, Angiotensin; ACE, Angiotensin converting enzyme; aP < 0.05 compared to CG in the normotensive sample, bP < 0.05 compared to NP in the normotensive sample, cP < 0.05 compared to DUP in the normotensive sample*.

When the analyses were performed by subgroups, CG and NP demonstrated overweight and obese phenotypes, respectively, while DUP showed a normal classification in the Normotensive subgroup. On the other hand, only NP was classified as overweight in the Hypertensive subgroup. Regarding blood pressure values, Normotensive and Hypertensive subgroups showed values according to the proposed cut-offs (Corona et al., 2014). Two-way ANOVA demonstrated elevated SBP in all Hypertensive groups when compared to Normotensive groups.

### Blood pressure in the overall sample

**Figure 3:**
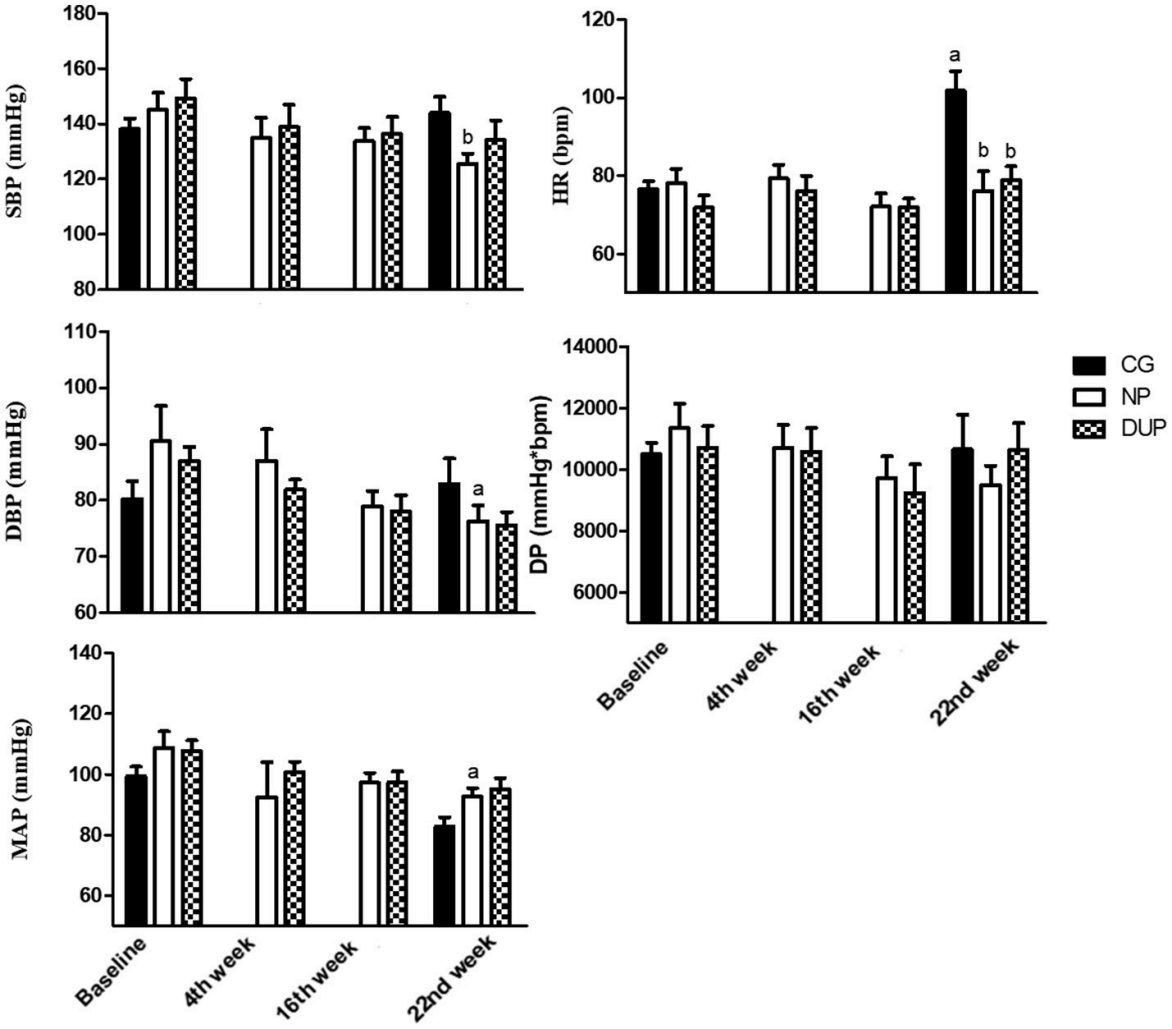
Hemodynamic parameters of experimental groups in the overall sample. Values expressed as mean ± SD.NP, Non-periodized; DUP, Daily undulating periodization; CG, Control group; SBP, Systolic blood pressure; DBP, Diastolic blood pressure; MAP, Mean arterial pressure; HR, Heart rate; DP, Double product; a*P* < 0.05 vs. Baseline; b*P* < 0.05 vs. CG in the 22nd week. An ANOVA [3 × 2, groups × time] was used to examine differences between NP, DUP and CG pre and post-exercise. An ANOVA [2 × 4, groups × time] was used to examine differences between NP and DUP over the 4 measurement moments (Baseline, 4th week, 16th week, and 22nd week). Dunnet was performed as the *post-hoc* test.

Figure 3 shows the hemodynamic parameters before (i.e., Baseline), during (i.e., 4th week and 16th week), and after (i.e., 22nd week) the experimental protocol in the whole sample. DBP and MAP were significantly reduced after NP. However, no significant alterations were observed in DUP. In turn, CG presented higher HR values when compared to Baseline, NP, and DUP on the 22^nd^ week. The ES classification of SBP (from small to moderate), DBP, and DP (from trivial to small in both) increased linearly in NP. A similar pattern was seen in SBP (from trivial to small) and DBP (from small to moderate) of DUP. In CG, a moderate classification was attributed to HR.

### Blood pressure in the hypertensive and normotensive sample

**Figure 4:**
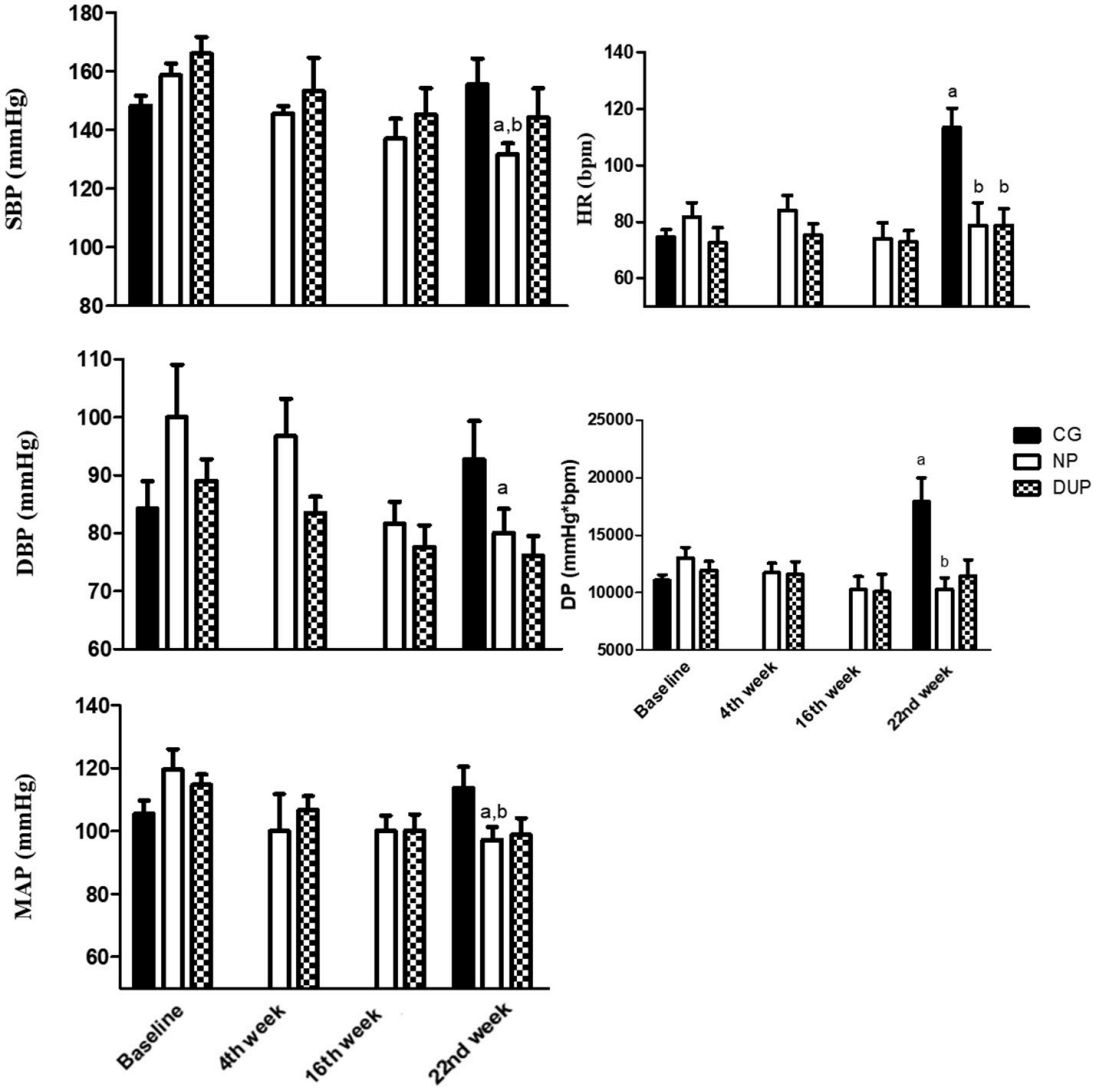
Hemodynamic parameters of experimental groups in the hypertensive subsample. Values expressed as mean ± SD.NP, Non-periodized; DUP, Daily undulating periodization; CG, Control group; SBP, Systolic blood pressure; DBP, Diastolic blood pressure; MAP, Mean arterial pressure; HR, Heart rate; DP, Double product; a*P* < 0.05 vs. Baseline; b*P* < 0.05 vs. CG in the 22nd week. An ANOVA [3 × 2, groups × time] was used to examine differences between NP, DUP and CG pre and post-exercise. An ANOVA [2 × 4, groups × time] was used to examine differences between NP and DUP over the 4 measurement moments (Baseline, 4th week, 16th week, and 22nd week). Dunnet was performed as the *post-hoc* test.

**Figure 5:**
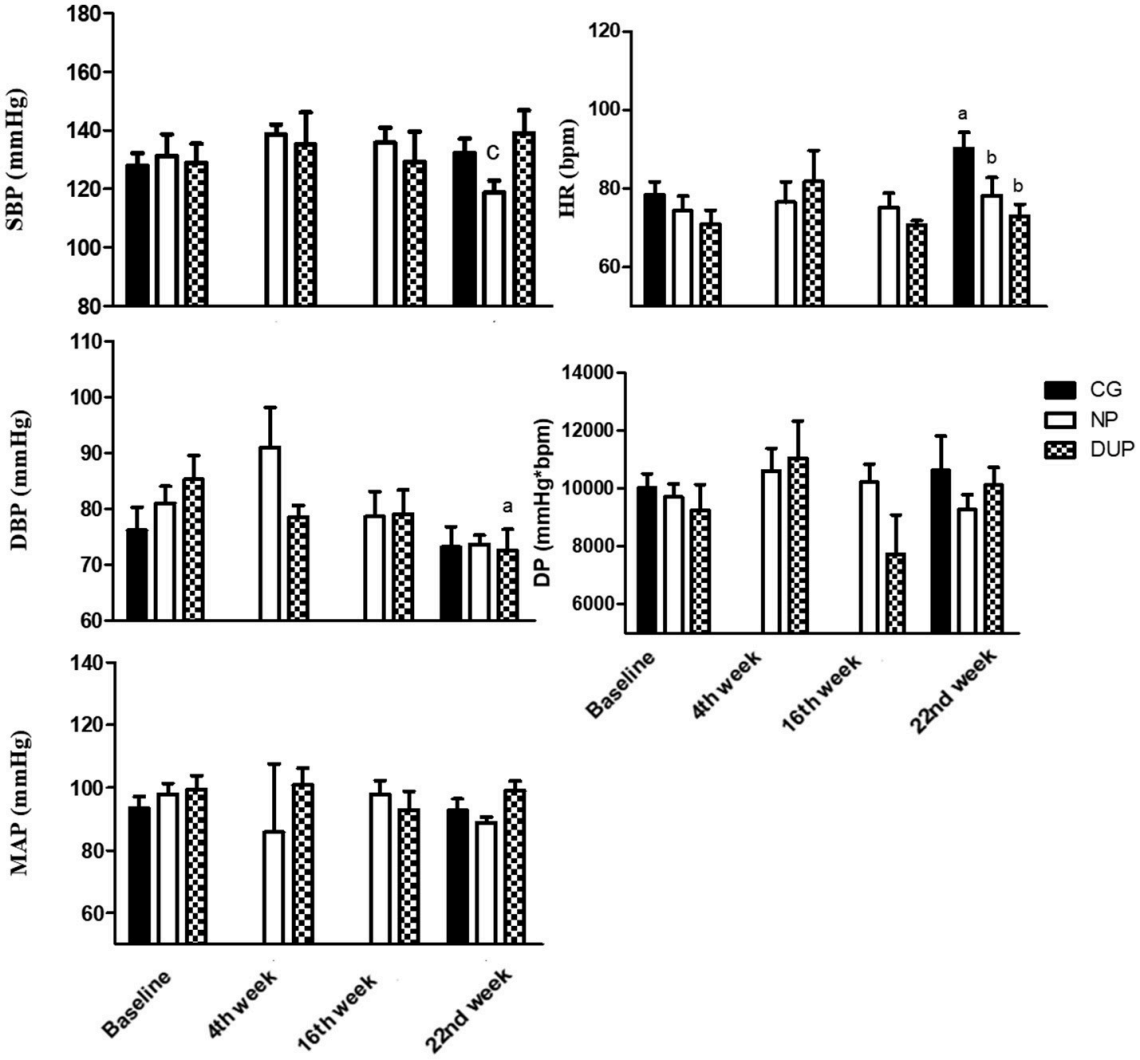
Hemodynamic parameters of experimental groups in the normotensive subsample. Values expressed as mean ± SD.NP, Non-periodized; DUP, Daily undulating periodization; CG, Control group; SBP, Systolic blood pressure; DBP, Diastolic blood pressure; MAP, Mean arterial pressure; HR, Heart rate; DP, Double product; a*P* < 0.05 vs. Baseline; b*P* < 0.05 vs. CG in the 22nd week; c*P* < 0.05 vs. DUP in the 22nd week. An ANOVA [3 × 2, groups × time] was used to examine differences between NP, DUP and CG pre and post-exercise. An ANOVA [2 × 4, groups × time] was used to examine differences between NP and DUP over the 4 measurement moments (Baseline, 4th week, 16th week, and 22nd week). Dunnet was performed as the *post-hoc* test.

When the analyses were performed by subgroups (Figures 4, 5), NP training demonstrated further benefits in the Hypertensive sample (Figure 4), since a significant decrease in SBP, DBP, and MAP was observed on the 22nd week. On the other hand, a significant increase in HR and DP was demonstrated by CG. DUP did not cause significant alterations in the hypertensive sample. When the comparisons were performed among the groups, lower SBP, MAP, HR, and DP were observed in NP, as well as a lower HR in DUP, when compared to CG on the 22nd week. Evaluations in the Normotensive sample (Figure 5) demonstrated that SBP values were lower in NP than in DUP. However, DUP, but not NP, was effective to decrease DBP values. As observed in the overall sample, HR values were elevated in CG when compared to Baseline, NP, and DUP on the 22^nd^ week. In CG, HR values of Hypertensive and Normotensive samples were classified as large and moderate, respectively. In the Hypertensive subgroup, ES classification was in concordance with the hypothesis test, because SBP reached a Large classification in NP. Moreover, as was observed in the overall sample, a linear increase in ES classification was observed for SBP, DBP, MAP, and DP in NP, as well as in DBP and MAP in DUP group; the highest ES classification was showed when *P* < 0.05. Regarding the Normotensive sample, comparisons between NP and DUP did not confirm ANOVA results because the trained groups showed small and moderate classifications on SBP and DBP values, respectively. However, the highest ES classification on DBP was reached by the NP. Lastly, the exercise groups demonstrated lower classifications on HR than CG.

### Biochemical analysis

**Figure 6:**
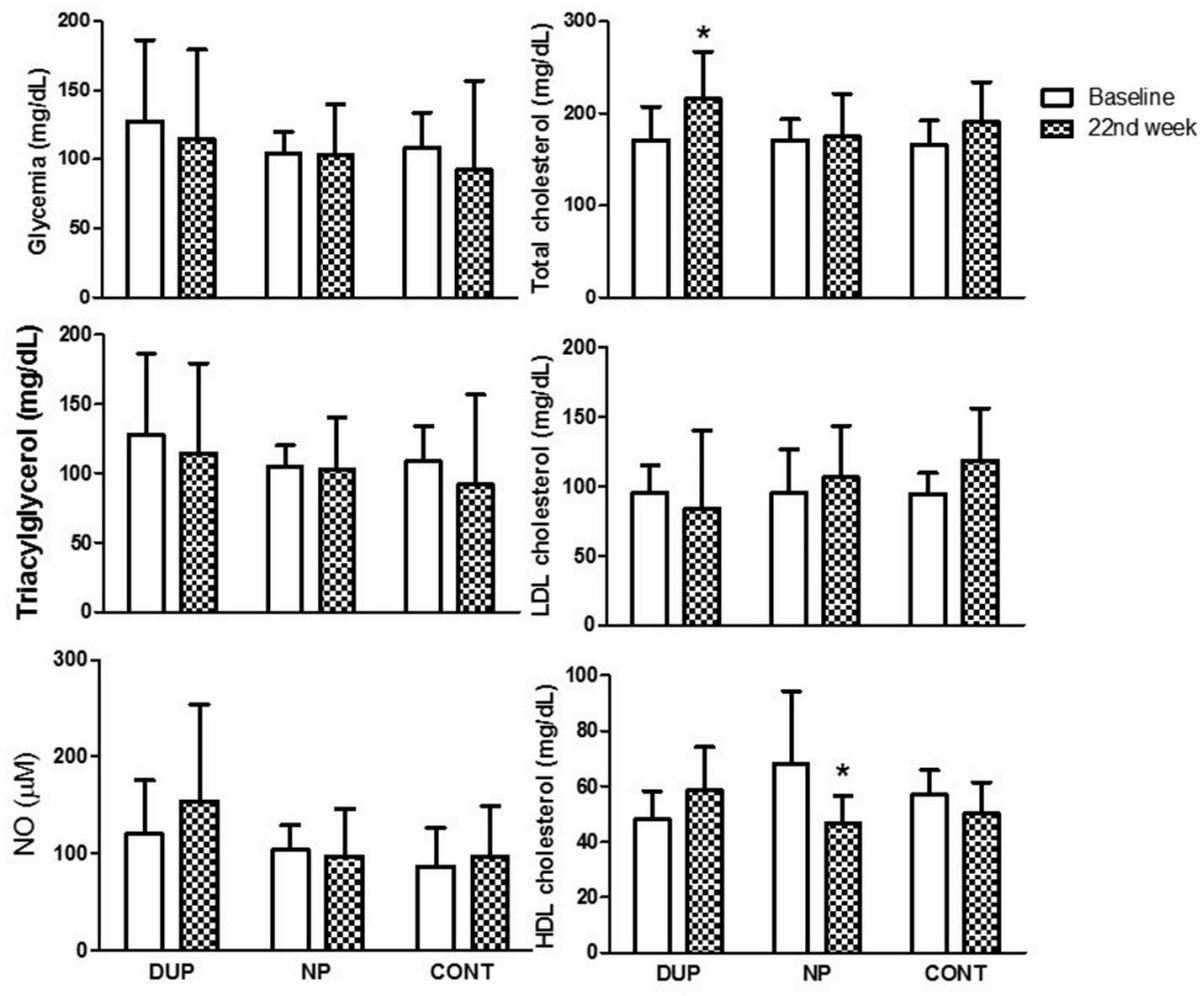
Biochemical parameters of experimental groups. Values expressed as mean ± SD.NP, Non-periodized; DUP, Daily undulating periodization; CG, Control group; NO, Nitric oxide; LDL, Low-density lipoprotein; HDL, High-density lipoprotein. An ANOVA [3 × 2, groups × time] was used to examine differences between NP, DUP, and CG pre and post-exercise.

Figure 6 shows the biochemical analysis before and after the protocols. DUP demonstrated a significant increase in total cholesterol levels after 22 weeks of RT, which was accompanied by a small ES classification. In turn, HDL cholesterol was decreased in the NP group (Cohens'd*:* small).

## Discussion

The main findings of the present study indicate that a 22-week NP exercise training program reduces DBP and MAP in older female volunteers. Moreover, although our study was not designed to evaluate the effects of DUP and NP on samples composed exclusively by hypertensive and normotensive volunteers, we observed further benefits on blood pressure when the sample was divided according to HTN, since hypertensive patients showed a significant reduction in SBP, DBP, and MAP after NP exercise training, but not after DUP. On the other hand, a decreased DBP was only observed after DUP in the normotensive group. It should be stressed, that both NP and DUP programs presented lower HR values on the 22nd week in comparison to CG.

In the current study, NP caused a significant reduction in SBP and DBP in the overall (SBP = −19.5 mmHg [13.5%]; DBP = −14.2 mmHg [15.7%]), hypertensive (SBP = −27 mmHg [17%]; DBP = −20 mmHg [20.0%]) and normotensive samples (SBP = −12.6 mmHg [9.6%]; DBP = −8.0 mmHg [9.8%]). Although significant values were only observed in the hypertensive sample, ES evaluation indicated that NP results were better ranked in intra and intergroup comparisons when compared to DUP and CG.

These data are supported by previous research, which has shown that NP programs are effective to decrease blood pressure values in hypertensive (Terra et al., 2008; Moraes et al., 2012; Mota et al., 2013; Tomeleri et al., 2017) and normotensive (Gerage et al., 2013) subjects. Indeed, in a recent experiment, Tomeleri et al. (2017) observed reduced blood pressure levels (SBP = −11%; DBP = −10%) in hypertensive older women who were submitted to a 12-week NP ST protocol. Similarly, Terra et al. (2008) and Moraes et al. (2012) observed decreased blood pressure values in hypertensive patients after NP programs.

Despite the similarities among the studies, it is necessary to highlight some bias associated with these previous trials, such as the short-term intervention period, samples composed of a mixture of middle-aged and older adults, and a high prevalence of patients with other comorbidities than HTN (e.g., diabetes mellitus type II). In addition, some exercise programs were prescribed based on the maximum number of repetitions that the volunteer could reach, which does not bring an attractive cost-benefit because blood pressure values increase progressively in these regimes of exercise, reaching the highest level during the last repetition (MacDougall et al., 1985; Paulo et al., 2017).

On the other hand, in the present investigation, we observed that older women submitted to a 22-week moderate intensity NP exercise training program demonstrated a greater decrease on hemodynamic parameters when compared to the aforementioned studies, regardless of the elevated intensity proposed by some of the studies. Nevertheless, it should be stressed that the main positive effects of the present study were observed in NP since DUP did not cause significant reductions in blood pressure values of hypertensive patients. Unfortunately, this is the first study that investigated the impact of a chronic DUP composed of ST and PT sessions, which severely limits our discussion.

It is well-accepted, that the chronic adaptations presented by the cardiovascular system in response to physical exercise may be due to the sum of the acute effects (Terra et al., 2008; Moreira et al., 2016). In this sense, an increasing number of studies have shown that a session of low-to-moderate load power exercise may cause post-exercise hypotension (PEH) in hypertensive older women (Coelho-Júnior et al., 2017a; Coelho Junior et al., 2017b). In addition, Arazi et al. (2013) and Ramírez-Campillo et al. (2016) demonstrated that PEH occurred independently of power exercise intensity (Arazi et al., 2013; Ramírez-Campillo et al., 2016), which is an important practical aspect for the prescription of exercise in hypertensive subjects. Therefore, in our point of view, these data suggested that exercise intensity does not play a key role in the hemodynamic adaptations in response to PT, leading us to combine low-to-moderate PT and traditional RT in a DUP program.

However, it should be stressed that the findings of Arazi et al. (2013) and Ramírez-Campillo et al. (2016) were based on young, physically-active normotensive volunteers, and in a plyometric training, which does not seem to be attractive to older adults since the osteoarticular apparatus of these patients presents a reduced functioning and plyometric training is dependent on the reactive properties of muscle. Moreover, because our program of PT was based on loads of ~50% 1RM, based on Day et al. (2004), it is possible to hypothesize that the exercise intensity proposed to PT did not provoke the minimum effort necessary to decrease blood pressure in hypertensive individuals. These inferences are supported by previous research, since most exercise training protocols were performed at low to moderate intensities, with loads close to 60% 1RM, and an exponential increase in training load across the exercise program (Terra et al., 2008; Mota et al., 2013).

Indeed, the lowest training load used in the exercise training protocols mentioned in the present study was 60% 1RM (Terra et al., 2008; Moraes et al., 2012; Mota et al., 2013), which represents a moderate intensity (Garber et al., 2011). However, the load was increased monthly until 80% 1RM (Terra et al., 2008; Mota et al., 2013)—vigorous intensity. Findings from studies that investigated the acute effects of resistance exercise on blood pressure have been demonstrating a similar phenomenon (Figueiredo et al., 2015). Therefore, it is possible to infer that a minimum intensity (≥60%1RM) seems to be necessary to elicit cardiovascular responses. Unfortunately, the evidence is scarce in relation to this specific issue, and future studies should be designed to allow better inferences.

It is noteworthy that the findings of the current study seem to have great practical application because a slight decrease in SBP and DBP reduces cardiovascular and cerebrovascular risk (Antonakoudis et al., 2007). Furthermore, CG demonstrated an exponential increase in HR over the 22 weeks, which was not observed in both exercise groups, indicating a cardioprotective role of NP and DUP programs. Several biological mechanisms may underlie the cardioprotective benefits observed in HR after NP and DUP, such as an ameliorated autonomic balance and an increased anti-inflammatory milieu mediated by myokines (to review see Fiuza-Luces et al., 2018). Once these possible mechanisms were not investigated in the present study, future studies should consider their assessment.

Moreover, there is no consensus regarding the effects of RT on NO bioavailability. In fact, acutely, we observed elevated salivary NO levels in response to the exercise programs used in the current study (Coelho-Júnior et al., 2017a). However, chronically, this phenomenon was not reported by Moraes et al. (2012) while Tomeleri et al. (2017) indicated NO as the main responsible for the reduced blood pressure levels observed in their study. The inconsistencies between these findings may be a function of the different exercise training designs, assay methods (e.g., Griess, NO analyzer), type of biological material evaluated (i.e., saliva, blood), and the number of morbidities associated with HTN, given that a subgroup of volunteers of the present study and from Moraes et al. (2012) were only hypertensive; whereas Tomeleri et al. (2017) recruited patients with diabetes, dyslipidemia, and fibromyalgia. When baseline data was confronted, for example, was possible to observe different NO levels at baseline (120 μM; Moraes et al. (2012), 26 μM; Tomeleri et al. (2017), 5.4 μM). Accordingly, several variables associated or not with exercise training prescription appear to influence NO levels and more controlled studies are still necessary.

Interestingly, although volunteers were asked not to change their diet or food habits during the experimental period, elevated total cholesterol and reduced HDL cholesterol levels were observed on the 22nd week in DUP, and NP, respectively, when compared to Baseline. An altered caloric intake may be responsible for this phenomenon, as well as for the lack of alterations in NO bioavailability since cholesterol levels are positively associated with the atherosclerotic process, decreasing NO levels (Cornelissen and Smart, 2013). Nevertheless, we should assume that more studies confirming the reliability of salivary NO as a method to assess vascular NO are still necessary. Since NO availability was unchanged in the groups, other mechanisms are responsible for the beneficial effects observed after NP exercise training, such as autonomic modulation (Shimojo et al., 2015; da Palma et al., 2016; Trevizani et al., 2017). Other limitations of the present study—in addition to the absence of the evaluation of the autonomic cardiac control—should be mentioned to collaborate with better inferences about the data, such as the lack of dietary control and the inclusion of individuals regardless of BMI.

It is worth mentioning that our sample size may limit the investigation of subgroups (i.e., normotensive and hypertensive), causing inconsistent results between ES and P-value. Indeed, our experimental design was based on 3, and not 6 groups, as stated in the statistical analyses. In this sense, inferences about the findings of our subgroup analyses should be taken carefully. Another important limitation of the present study is the lack of a control hemodynamic evaluation performed a few weeks before the baseline evaluation, which is helpful to avoid bias and the influence of external factors. Finally, we proposed PT as a safer kind of RT than traditional ST. However, we do not have enough data to support this hypothesis. Therefore, future studies with larger sample sizes, other periodization designs, sessions of PT performed at higher intensities, and more detailed evaluations should be performed to confirm the current findings.

## Conclusion

In conclusion, our findings indicate that a 22-week NP RT program causes beneficial effects on hemodynamic parameters of older women.

## Author contributions

HC-J conception and design, analysis, and interpretation of data, drafting the article and analysis and interpretation of data for important intellectual content; final approval of the version to be submitted. BR drafting the article and analysis and interpretation of data for important intellectual content; final approval of the version to be submitted; NC, IG, MC, and RB analysis, acquisition, and interpretation of data, critical review for important intellectual content. JS, EC, and RA conception and design, analysis, and interpretation of data, drafting the article and analysis and interpretation of data for important intellectual content; final approval of the version to be submitted. MU conception and design, analysis and interpretation of data, drafting the article and analysis and interpretation of data for important intellectual content; final approval of the version to be submitted.

### Conflict of interest statement

The authors declare that the research was conducted in the absence of any commercial or financial relationships that could be construed as a potential conflict of interest.
